# Study of Mental Activity and Regular Training (SMART) in at risk individuals: A randomised double blind, sham controlled, longitudinal trial

**DOI:** 10.1186/1471-2318-11-19

**Published:** 2011-04-21

**Authors:** Nicola J Gates, Michael Valenzuela, Perminder S Sachdev, Nalin A Singh, Bernhard T Baune, Henry Brodaty, Chao Suo, Nidhi Jain, Guy C Wilson, Yi Wang, Michael K Baker, Dominique Williamson, Nasim Foroughi, Maria A Fiatarone Singh

**Affiliations:** 1 School of Psychiatry, University of New South Wales, Randwick NSW 2031, Australia; 2 Brain and Aging Research Program, University of New South Wales, Randwick NSW 2031, Australia; 3 Regenerative Neuroscience Group, School of Psychiatry, University of New South Wales, Randwick NSW 2031, Australia; 4 Neuropsychiatric Institute, Prince of Wales Hospital, Randwick NSW 2031, Australia; 5 Balmain Hospital, Balmain NSW 2041, Australia; 6 Royal Prince Alfred Hospital, Camperdown NSW 2050, Australia; 7 Discipline of Psychiatry, School of Medicine, University of Adelaide, Adelaide SA 5005, Australia; 8 Primary Dementia Collaborative Research Centre, University of New South Wales, Randwick NSW 2031, Australia; 9 Exercise Health and Performance Faculty Research Group, Sydney Medical School, The University of Sydney, Lidcombe NSW 2141, Australia; 10 Hebrew Senior Life, Boston, MA, and Jean Mayer USDA Human Nutrition Research Center on Aging at Tufts University, Boston 02130, MA, USA

## Abstract

**Background:**

The extent to which mental and physical exercise may slow cognitive decline in adults with early signs of cognitive impairment is unknown. This article provides the rationale and methodology of the first trial to investigate the isolated and combined effects of cognitive training (CT) and progressive resistance training (PRT) on general cognitive function and functional independence in older adults with early cognitive impairment: Study of Mental and Regular Training (SMART). Our secondary aim is to quantify the differential adaptations to these interventions in terms of brain morphology and function, cardiovascular and metabolic function, exercise capacity, psychological state and body composition, to identify the potential mechanisms of benefit and broader health status effects.

**Methods:**

SMART is a double-blind randomized, double sham-controlled trial. One hundred and thirty-two community-dwelling volunteers will be recruited. Primary inclusion criteria are: at risk for cognitive decline as defined by neuropsychology assessment, low physical activity levels, stable disease, and age over 55 years. The two active interventions are computerized CT and whole body, high intensity PRT. The two sham interventions are educational videos and seated calisthenics. Participants are randomized into 1 of 4 supervised training groups (2 d/wk × 6 mo) in a fully factorial design. Primary outcomes measured at baseline, 6, and 18 months are the Alzheimer's Disease Assessment Scale (ADAS-Cog), neuropsychological test scores, and Bayer Informant Instrumental Activities of Daily Living (B-IADLs). Secondary outcomes are psychological well-being, quality of life, cardiovascular and musculoskeletal function, body composition, insulin resistance, systemic inflammation and anabolic/neurotrophic hormones, and brain morphology and function via Magnetic Resonance Imaging (MRI) and Spectroscopy (fMRS).

**Discussion:**

SMART will provide a novel evaluation of the immediate and long term benefits of CT, PRT, and combined CT and PRT on global cognitive function and brain morphology, as well as potential underlying mechanisms of adaptation in older adults at risk of further cognitive decline.

**Trial Registration:**

Australia and New Zealand Clinical Trials Register (ANZCTR): ANZCTRN12608000489392

## Background

With a forecast 100 million persons with dementia by 2050, this disorder presents a major challenge to sufferers, their caregivers, and the health care system, and delay of disease onset and progression is amongst the most pressing challenges for medical research [1]. A five-year delay in dementia onset and progression could halve disease prevalence [2] and would have a significant impact on disease burden. The efficacy of pharmacological treatments to date have been limited to symptom control [3] and have not been effective in reducing disease onset, and so non-pharmacological preventative interventions are of great interest.

There is strong evidence from cross-sectional and prospective cohort studies that participation in mentally and physically stimulating activities is associated with decreased dementia prevalence and/or incidence [4-9].

Experimental trials indicate that cognitive training can significantly improve performance in healthy adults on a range of cognitive tasks, with an average moderate effect size (ES) of 0.6 [10-13]; and that exercise interventions of as little as one week of aerobic exercise can result in improved memory, attention, and reaction time [14]. Sustained improvements, particularly in executive function, have been shown after aerobic training (ES = 0.41), combined aerobic and resistance training (ES = 0.59), and resistance training alone (ES = 0.53), even after exercise was withdrawn in some cases [15].

Two studies to directly compare single and combined physical and mental exercise found effect sizes across a range of cognitive outcomes to be much larger in the combined condition [12,16]. Both of these studies had design flaws, including very small sample sizes [16] and high dropout rates [12], limiting conclusions. Therefore, a robustly designed trial is required to investigate the comparative benefits of isolated and combined physical and mental training.

The mechanisms of benefit from physical and mental interventions are not clear, it has been postulated that physical and mental activity may therefore have potential to stimulate plasticity of the brain and possibly reduce dementia onset. Animal studies have demonstrated a range of positive neurobiological outcomes, including decreased inflammatory cytokines, decreased cortisol response to stressors, increased insulin-like growth factor-1 (IGF-1) into the brain, increased cerebral blood flow and angiogenesis, and increased hippocampal volume, brain derived neurotrophic factor (BDNF), neurogenesis, and synaptic density after memory-enhancing cognitive and exercise training [7,17]. Human data are more limited, but observations of responses to training have included increased blood flow, aerobic capacity, and region brain volume after behavioural and aerobic training [18,19] and improved brain chemistry using magnetic resonance spectrometry (MRS) in our pilot work with cognitive training [20]. There are fewer human data available on the possible cognitive-enhancing mechanisms of resistance training, with findings of no changes in BDNF [21], and increases in IGF-1 [22]. Animal and human exercise trials indicate that exercise may improve brain function via two pathways; directly through the induction and regulation of growth factors (e.g., BDNF, IGF-1), and/or indirectly via the modulation of systemic inflammation [7]. However, rigorous clinical trials investigating the central and peripheral synergistic benefits of exercise for improved brain function are lacking [7]. Consequently the Study of Mental Activity and Regime Training trial (SMART) was designed and implemented to examine the isolated and combined benefits of cognitive training and resistance training, and to provide novel, comprehensive data on possible proposed links between cognitive improvement and brain and whole-body-adaptation to resistance and cognitive training.

**Figure 1:**
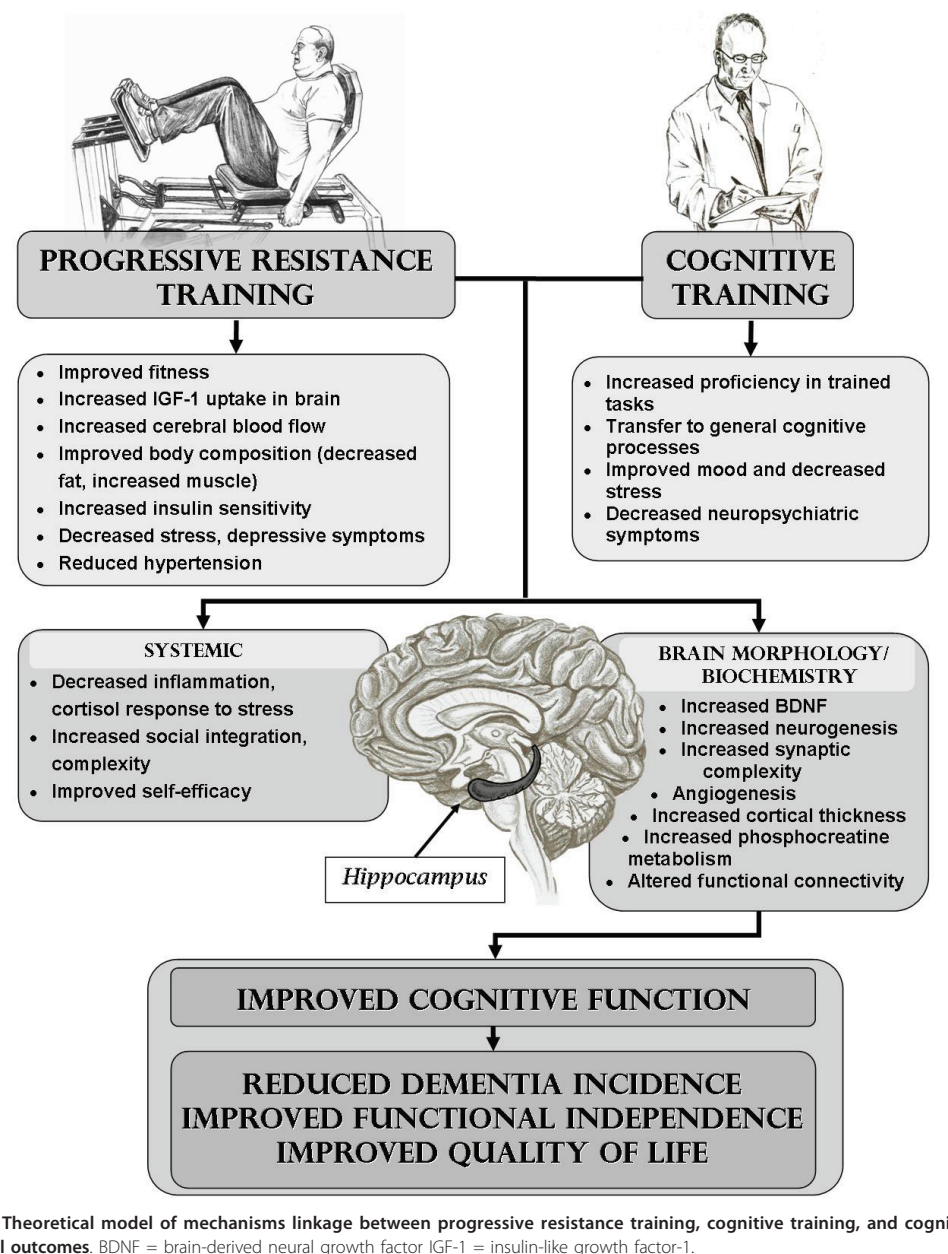
**Theoretical model of mechanisms linkage between progressive resistance training, cognitive training, and cognitive and functional outcomes**. BDNF = brain-derived neural growth factor IGF-1 = insulin-like growth factor-1.

Most cognitive and exercise training trials have targeted healthy, cognitively intact adults. The most vulnerable individuals at highest risk for cognitive decline, however, are those with early cognitive impairment and co-morbid diseases such as cardiovascular disease, type 2 diabetes, obesity, and hypertension (i.e. metabolic syndrome). We are therefore deliberately excluding high functioning volunteers and targeting a highly clinically relevant population, with evidence of early cognitive impairment and various cardiovascular risk factors. These individuals may not be capable of the moderate or high intensity aerobic training that has been shown to be effective in animal and human trials. Resistance training, which has a larger effect size in the literature (0.53) than isolated aerobic training (0.41), and comparable to combined aerobic/resistance training (0.59), may be a more realistic exercise option in this cohort, as it is more feasible in elders with frailty and mobility impairment, thus having the potential for long-term adherence. We [23] and others have shown that resistance training results in many beneficial adaptations in older adults that may be relevant to the mechanisms underlying its putative cognitive benefits. These adaptations (see Figure 1), many of which will be studied in this proposal (particularly changes in fitness, inflammation, and body composition) would not be anticipated after exposure to cognitive training alone, consequently the SMART trial will enable investigation of the efficacy of combining these two distinctly different training paradigms.

The SMART trial is a long term study that will measure evidence of both immediate and sustained benefits of training, one year after withdrawal of active treatment. In addition to the selection of general cognitive, functional, physical, mood symptoms and quality of life outcome measures will also be assessed to identify the extent of transfer of benefits of our interventions.

### Objectives and Hypothesis

The primary objective of the SMART trial is *to determine whether cognitive, physical or combined cognitive and physical training can prevent or slow cognitive and functional decline in vulnerable older adults at high risk of dementia*. Our secondary aim is to explore adaptation to these two interventions in the brain, as well as identify potential mechanisms of benefit, in particular modulation of cardiovascular risk profile, systemic inflammatory cytokines, growth factors, fitness levels, and body composition.

#### Primary Hypotheses

1. Six months of supervised cognitive training (CT) will significantly improve cognitive function, as assessed by the Alzheimer's Disease Assessment Scale Cognitive subscale (ADAS-Cog) [24,25], and independence of function as assessed by the Bayer Informant -Activities of Daily Living (BIADL) [26] at both 6- and 18- month follow-up, relative to a sham training control condition.

2. Six months of supervised high intensity progressive resistance training (PRT) will significantly improve cognitive function, as assessed by the ADAS-Cog, and independence of function as assessed by the Bayer Informant -Activities of Daily Living (BIADL), at both 6- and 18-month follow-up, relative to a sham training control condition.

3. The combination of CT and PRT will be significantly superior to either intervention in isolation for cognitive and functional benefits.

#### Secondary Hypotheses

1. All active training interventions will improve brain morphology and biochemistry compared to the sham control condition, as defined by: increased hippocampal volume (mm^3^) by MRI scanning; positive localised Voxel-Based Morphometry (VBM) brain changes (z-score relative change); decreased whole brain volume of White Matter Hyper-intensities (WMHs) (mm^3^); and lead to beneficial hippocampal and posterior cingulate MRS metabolite changes (% increase in N-acetylaspartate, and increase in phosphocreatine metabolites).

2. All active training interventions will improve secondary cognitive outcomes, in the domains of attention, memory, fluency, and executive function, relative to the sham control condition, and combined training will be superior to either single intervention.

3. All active training interventions will maintain global clinical impression scores, as defined by the Clinical Dementia Rating (CDR) scale, relative to the sham control condition.

4. PRT exercise will preferentially decrease inflammatory markers, insulin resistance, and central adiposity and increase fitness (strength and aerobic capacity), muscle mass, and functional mobility, compared to either cognitive or sham control condition.

5. Cognitive and physical training will produce positive effects on psychological health and quality of life above and beyond the non-specific effects seen after sham control condition.

## Methods

### Study Design and Setting

The SMART trial is a longitudinal double-blind, sham training-controlled, randomized clinical trial adhering precisely to CONSORT guidelines http://www.consort-statement.org for the conduct and reporting of clinical trials, as extended to non-pharmacological interventions [27]. Ethical approval was obtained from the Sydney South West Area Health Service (HREC Ref.08/RPAH/106), University of Sydney Human Research Ethics (HREC: 06-2008/11094), University of New South Wales (HREC: 08152), and signed informed consent was obtained from all participants. Participants are from the greater Sydney metropolitan area, and the study is conducted at Cumberland Campus of the University of Sydney in Lidcombe NSW Australia. MRI scans are performed at the Clinical Research Imaging Centre in Randwick NSW Australia.

### Study Population and Eligibility Criteria

Participants are community-dwelling persons aged 55 or above, with primary inclusion criteria being self-reported memory complaint, a Clinical Dementia Rating (CDR) [28] of ≤ 1.0; Mini-Mental Status Examination (MMSE) [29] score of 23-29; and willing to have multiple cognitive, physical and imaging assessments over 18 months. Complete inclusion and exclusion criteria are listed in Table 1.

**Table 1:** Inclusion and exclusion criteria for the SMART trial

Inclusion criteria	Exclusion criteria
Age ≥ 55 Competency in English sufficient for assessment and training Community-dwelling MMSE^ 23-29 CDR^^ ≤ 1.0 TICS ^#^< 30 No unstable disease precluding planned exercise* Absence of significant cognitive decline in 5 years Absence of known organic or psychiatric condition affecting cognition Able to see and hear sufficiently to participate in planned physical and computer-based cognitive training	Unstable medical condition* Participation in any cognitive training activity Participation in > 150 min/wk of moderate or greater intensity planned exercise of any kind Rapidly progressive or terminal illness Pre-existing diagnosis of dementia Psychotic illness (DSM-IV)** Degenerative neurological disorder Diagnosis of stroke or TIA^+ ^within last 12 months, stroke with residual neurological deficit, two or more strokes or TIAs at anytime, One stroke or TIA with residual deficits that preclude participation. TBI ^± ^within past year, or with residual deficits that preclude participation. Diagnosis of depression (DSM-IV) GDS^++ ^>9 or current treatment with antidepressant medications, greater than 3 episodes of depression in the last 5 years ("episode": requiring treatment), > 10 episodes requiring treatment over lifetime, past suicide attempts, current bipolar diagnosis and treatment, > 3 past episodes requiring treatment in last 5 years. Current alcohol or drug abuse (DSM-IV) Unrepaired abdominal or other known aneurysm Myocardial infarction or cardiac surgery within past 6 months NYHA Class IV CHF^±±^ Unstable angina or uncontrolled malignant arrhythmias at rest or on exercise stress testing Recent retinal haemorrhage or detachment/proliferative retinopathy Seizures (>2 in past 12 months)

* Examples of unstable conditions include uncontrolled arrhythmias, hypertension, hyperglycemia, symptomatic enlarging hernia, acute pulmonary embolism, deep venous thrombosis, recent or unstable fracture, inflammatory or traumatic joint injuries, etc. Such individuals may become eligible if medical or surgical treatment stabilizes their condition.

^ MMSE Mini Mental Status Examination ^27^

^^ CDR Clinical Dementia Rating ^26^

^#^TICS Telephone Interview for Cognitive Status -Modified ^30^

**DSM-IV Diagnostic and Statistical Manual of Mental Disorders 4^th ^edition

^+ ^TIA Trans Ischemic Attack

^± ^TBI Traumatic brain Injury

^++ ^GDS Geriatric Depression Scale [49]

^±±^NYHA Class IV CHF New York Heart Association Class IV Class of Heart Failure indicating symptoms evident at rest with any physical activity increasing symptoms, precluding ability to complete physical activity and causing severe limitations.

### Recruitment

Participants are to be recruited from October 2008 until December 2011 from media publicity on state radio, advertisements in local newspapers and businesses, a mail campaign using the electoral roll, contact with participants from previous studies who provided consent for such contact, general practitioner lists, aged care and health service facilities, community programs for seniors and word of mouth.

#### Sample size estimates

Sample size estimates (alpha 0.05, beta 0.20) are based on planned comparisons for the main effects of PRT and CT, as well as the effect of combined training vs. either intervention in isolation on our primary outcome: global cognitive function as assessed by ADAS-Cog. The assumptions are as follows: our meta-analyses [30] and review of published RCTs in older adults [15] reveal Effect Sizes (ES) for a range of cognitive outcomes of approximately 0.60 for cognitive training, 0.59 for aerobic/resistance training, and 0.53 for resistance training, compared to 0.15 for control groups. However, as we are enrolling a cohort with early cognitive impairment, we anticipate a decline of approximately this magnitude (ES = 0.15) over 12 months in our sample, so that the sham control condition would merely offset that decline (ES = 0.0). Thus, we have conservatively powered the study to show an ES of 0.53 for the main effects of both CT and PRT vs. control (n rounded up to 30/group × 4 = 120 required for 4-cell factorial design).

The only two published studies of *combined mental and physical training *[12,16] showed average ES = 0.94 for combined training compared to mental training alone and ES = 1.27 for combined training compared to exercise training alone. Therefore, we have ample power (99.7%) to find a difference of this magnitude between our combined training (n = 30) and isolated training groups (n = 30). Reported dropout from drug trials in MCI is 28% [31] however our experience in fully supervised training of older adults with frailty/chronic disease dropout averages 10-15% over 12 months. Therefore, we will inflate sample size needs for approximately10% drop out rate to account for anticipated attrition (n = 132), and we will recalculate these sample size needs in interim analysis after the first 50 participants have completed 6 months intervention and revise ES and sample size needs if required.

#### Screening procedure

Potential participants undergo initial telephone interview and screening using the 13-item modified Telephone Interview for Cognitive Status (TICS-M) [32], inclusion score between 21-30/39 to exclude high cognitively functioning individuals. Health status and lifestyle behaviours are also assessed via telephone. Informants are interviewed using the Bayer-Activities of Daily Living (B-IADL) [26], Informant KATZ Index of ADL [33], and informant ratings of memory decline and concern. A subset of informants complete an in-person B-IADL form to validate the telephone version.

**Figure 2:**
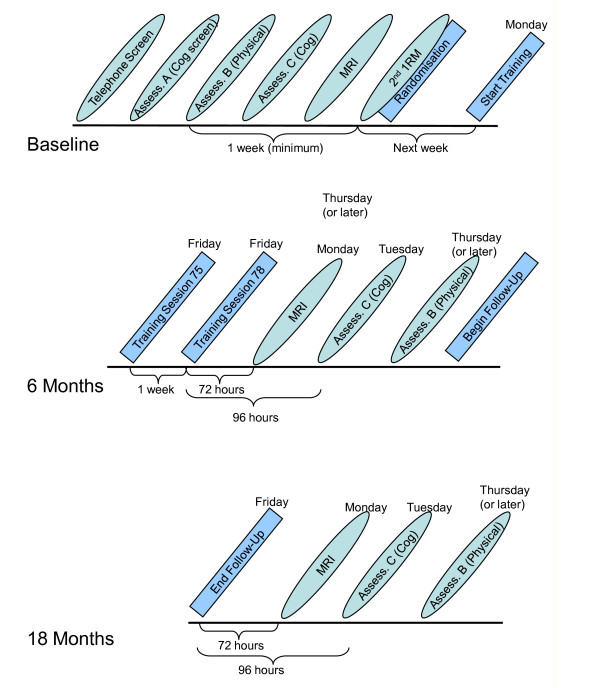
**SMART assessment schedule**.

Participants provide signed informed consent prior to completing a series of in-person screening assessments. A flow of assessment procedures is presented in Figure 2. A structured clinical interview including psychiatric screening is completed by a neuropsychologist, and CDR score is calculated prior to physician and physical screening. If eligible after physician screening, the remainder of the baseline physical performance testing is completed, followed by baseline cognitive tests and MRI scan. If following screening a participant was excluded for low vitamin D, acute illness, or abnormal stress test or raised blood pressure, he or she may enter the study following appropriate treatment and medical review. Participants are randomised at the completion of all baseline assessments.

### Randomisation, concealment, and allocation

**Figure 3:**
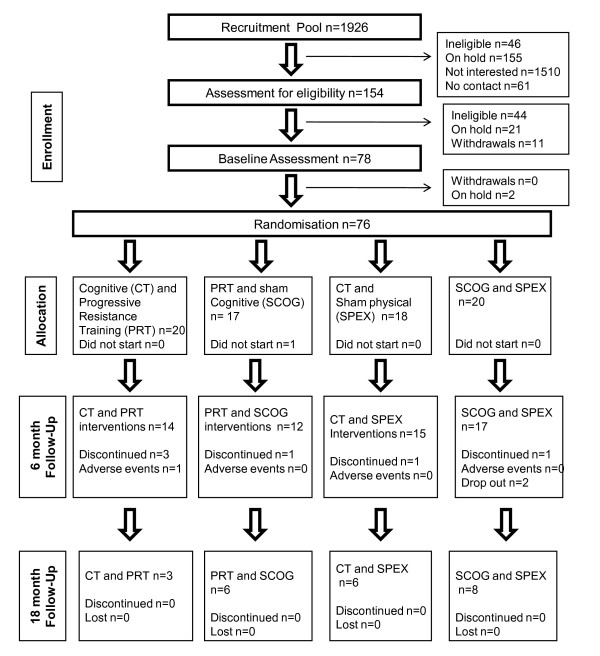
**Participant flow through SMART to date**.

A concealed, computer-generated sequence of randomly permuted blocks (block size = 8), stratified by gender and age, is generated by a statistician not otherwise involved in the study (http://www.randomization.com, created by Dr Gerard E. Dallal, Tufts University). Randomization occurs at the completion of the entire baseline assessment. Where randomization occurs in person, assignments will be placed in sealed opaque envelopes and delivered to subjects by the recruitment officer with subjects designated to 6 months of cognitive training, progressive resistance training, combined cognitive and progressive resistance training, or stretching and video-quiz control group in a 1:1:1:1 ratio. Flow of subjects through the study to date is presented in Figure 3.

### Stratification

Stratification by gender and age group (55-74; 75+) is carried out, in anticipation of the greater prevalence of women in the targeted cohort, and potential age effects on adaptation to training.

### Blinding

Subjects are informed that they will be randomly assigned to one of four treatment groups by the recruitment officer, and will be blinded to the investigators' hypothesis as to which is the preferred intervention arm. All groups will have an equal volume and frequency of contact with trainers over the 18 months of the study. All primary and secondary outcomes will be obtained and analyzed by blinded assessors on different days to the training programs.

### Interventions

All participants complete two group training sessions per week (total 26 weeks), under the supervision of trainers. Each session lasts 90 minutes and comprises approximately 45 minutes PRT or sham physical exercise (sham physical) and 45 minutes CT or sham cognitive exercise (sham CT). In order to take advantage of the enhanced attention and learning exhibited after an acute bout of exercise in both animal and human studies [34], but not enhance adaptation to sham cognitive training, PRT will proceed CT, and will follow sham CT.

Within each small group (maximum 10) participants follow the program tailored to their individual functioning level, with constant oversight by trainers. Make-up sessions are allowed for participants who miss CT and PRT sessions to achieve as close to 52 (26 × 2) sessions as possible within the 26 week intervention period. Each training group will have 1-2 trainers present at the session. The background of the trainers is in exercise physiology or physiotherapy, and the specific techniques of CT and PRT to be administered are learned from the investigators of this study (NG, MV, MFS).

Throughout the 18- month trial participants are provided with log books to document their social and recreational activities per day and are called weekly for telephone administration of a health status checklist. At the completion of the 6- month intervention participants are not given ongoing access to the training or advice as to what to do. Following assessments at 6 and 18 months participants receive a token reward (movie tickets or store voucher) for their participation.

#### Cognitive Training Intervention (+ Sham physical)

Cognitive training (CT) intervention involves computer-based multimodal and multi-domain exercises targeting memory, executive function, attention and speed of information processing. The training uses the COGPACK program [35], developed for neuro-rehabilitation and used in a previous research trial with MCI [36]. A total set of 14 exercises have been selected including six tasks of visual and verbal explicit memory ('Reading', 'Memory for names', 'Memory for shopping list', 'Memory for forms', 'Memory for route', 'Memory for traffic signs'), four tasks of executive function ('Anagrams', 'Sequence', 'Logic blocks', 'Logic') and four attention and speed tasks ('Reaction', 'Connect' UFOs' and 'Search'). The training schedule was pre programmed with 4 exercises being administered at each 45-minute training session. Training sessions are completed in a group setting with up to 10 computer work stations, and simple touch screens to avoid training difficulties in the computer-naïve.

#### Progressive Resistance Training (+ Sham cognitive)

Progressive resistance training (PRT) is supervised by experienced research assistants (exercise physiologists and physiotherapists) in a medically-supervised clinic (University of Sydney Health Sciences) at a ratio of 1 trainer for 4-5 subjects. The specifics of the high intensity training intervention are summarised in Table 2. Participants are progressed continuously throughout the 6-month intervention, guided by daily ratings of perceived exertion (15-18 on the Borg Scale [37] and 1RM's every 3 weeks to maintain intensity at 3% from 80 to 92% of current maximum capacity). Maximization of potential cognitive- enhancing effects of the PRT is supported by introduction of novel exercise after every 8 sessions and encouraging visualisation, counting out loud, and imagery of the muscle repetitions contracting during rest intervals.

**Table 2:** Progressive Resistance Training Methodology

	Exercise	Equipment	Frequency	Volume	Intensity progression
Routine	Seated Chest press	Digital K400 Keiser pneumatic resistance machines (Keiser Sports health Equipments, Inc. Fresno, CA)	2 sessions/wk	3 sets of 8 per session	80% of the most recently measured 1RM progressed each session s tolerated using RPE* 15-18 (approximately 3% per session)
	Seated leg press				
	Seated row				
	Standing hip abduction				
	Knee extension				

Novel	Lateral raise	Free weights (dumbbells)	4 weeks or 8 sessions	3 sets of 8 per session	15-18 RPE
	Hip flexion	Keiser			
	Calf raise	Keiser			
	Hip extension	Keiser			
	Bicep curls	Free weights (dumbbells)			

*RPE Ratings of perceived Exertion Borg Scale [37]

#### Combined CT and PRT

This group will receive both the cognitive training intervention and progressive resistance training intervention, delivered on the same day within 90-minute sessions.

#### Sham Cognitive and Sham Physical Exercise Control Group

In this group, subjects will receive versions of cognitive and physical exercise that are considered to be ineffective with regards to the cognitive, neurological and physical outcomes of this trial. The total session length will be 90 minutes, and all training will be supervised in groups of up to 10.

#### Sham Cognitive

Sham CT involves video exposure to a variety of general interest documentary topics, such as travel, culture, and history (National Geographic), and tailored to suit the audience and their expectation of training, are followed by a set of simple questions regarding the presented material. Previous CT trials used this type of active control condition [38] with minimal impact on cognitive outcomes.

#### Sham Physical

Sham physical exercise (Sham physical) will include stretching and seated calisthenics designed so as not to notably increase heart rate or aerobic capacity, improve balance, or enhance strength. No use of equipment and no progression will be included. This regime allows for maintenance of the double blind design as it is similar to what older adults anticipate receiving in senior group exercise classes. Furthermore, in contrast to aerobic activity, such a regimen has been shown recently to have no effects on brain volume in older adults [18].

### Adverse Events

Monitoring of adverse events will be achieved by weekly questionnaire/interview- and proxy information will be obtained whenever necessary to minimize missing data. Adverse events will include any exacerbation of underlying disease, or new onset musculoskeletal, cardiovascular, or metabolic abnormality attributed directly to study protocols.

Specific adverse events that will be routinely monitored include: falls, cardiac events during physical testing and exercise training (angina, arrhythmias, blood pressure excursions, clinically significant ECG changes); fatigue and muscle soreness or musculoskeletal injury after resistance or sham physical training; anxiety during MRI scan or cognitive or sham cognitive training; pain or injury related to movement of ferromagnetic devices, implants, shrapnel during MRI scan; and pain, bruising, or infection at the venipuncture site. In addition, subjects will be asked to report all changes in medication, health care professional visits, new diagnoses, acute illnesses, or any new symptoms.

### Outcome Measures

All outcome measures (see Tables 3, 4, 5, 6 and 7) will be administered by blinded assessors at baseline, 6 months and at 18 months follow-up. Each test is chosen because of excellent psychometric properties and minimal sensitivity to practice effects. Cognitive testing takes place in a fed state (after breakfast), and before any physical testing on that day to standardize known effects of fasting and acute exercise on cognitive performance.

**Table 3:** Primary and secondary cognitive and functional outcome measures

Outcome measure	Explanation	Description
Primary Cognitive	Alzheimer's Disease Assessment Scale (ADAS-Cog) [25]	This subscale of the ADAS, measures severity of cognitive dysfunction associated with Alzheimer's disease and is widely used in pharmacological studies of dementia and MCI [51]. The ADAS-Cog has excellent psychometric properties being valid and reliable, and is endorsed as a standard outcome measure [26].

Primary Functional	Bayer-Instrumental Activities of daily Living (B-ADL) [26,52]	The B-IADL initially developed for pharmaceutical clinical trials to assess deficits in the activities of daily life in community-dwelling individuals with MCI and response to pharmacological agents [26], is a 25-item informant or proxy questionnaire.

Secondary Cognitive	Mini-Mental State Examination (MMSE)[29]	Internationally known brief measure to screen for cognitive impairment [41], with valid and reliable quantitative assessment of severity of cognitive impairment, and is sensitive to changes in cognitive function over time [53].
	
	GP-Cog [54]	Six item self report scale identifying whether patients have greater difficulty functioning in 6 areas of daily life compared to their level of functioning 5-10 years earlier.
	
	Clinical Dementia Rating (CDR) [28]	A commonly used clinical tool for the global assessment of dementia severity, is completed by a clinician after synthesizing information obtained from the patient, informants and any other sources [28].

	Subjective Memory Complaint (SMC)	Eight questions were developed to measure SMC including type of memory difficulty, concern level, duration, comparison to peers, and reported by informant, meeting criteria for the assessment of SMC [55].

	Life Experience Questionnaire (LEQ)[19]	Questionnaire is a self report questionnaire examining the amount and quality of mental activity a person has engaged in over their life time [19].

	Matrices	Matrices, a perceptual subtest of the Wechsler Adult Intelligence Scale-III (WAIS-III) assesses executive functions and requires visual perception, organization, and synthesis of visual spatial information [56].

	Similarities	This verbal subtest from the WAIS-III is used to measure verbal conception formation and abstraction [56].

	Trail Making Test (TMT)[40] A and B	Trials A and B test speed of attention, sequencing and visual search, and includes a motor response component, whilst B also assesses mental flexibility, an executive function [57].

	Logical Memory	The Logical Memory subtest of the Wechsler Memory Scale 3^rd ^edition (WMS-III) is used to measure both immediate and delayed memory for verbal information.

	Benton Visual Retention Test (BVRT)[42]	This widely used visual memory test assesses visual perception and visual constructive abilities as participants are required to draw from memory simple designs [41].

	Symbol Digit Modalities Test (SDMT)	SDMT, first published in 1973 by Aaron Smith and subsequently revised in 1982[41] measures divided attention, visual scanning, tracking, and motor speed. It uses a substitution format presenting symbols with matching numbers, and participants are required to name the numbers corresponding to each given symbol.

	Category Fluency	Category Verbal Fluency measures verbal production of animal names from semantic memory [58].

	Controlled Oral Word Association Test (COWAT)	COWAT is a language based task assesses association fluency, and is often used as a measure of executive functioning. The most commonly used letters are F. A. S. or C. F. and L. based upon word prevalence rates [58].

	Memory Awareness Rating Scale-Memory Functioning (MARS-MF) [43]	The MARS-MF is an 11 item self report rating scale of everyday memory functioning. Ratings are made on a 0 - 4 scale where 0 = never and 4 = always, and is usually administered in an interview format [43].

**Table 4:** Secondary outcome measures continued: Psycho-social status

Outcome Measure	Name of scale	Description
Psycho-social	Geriatric Depression Scale (GDS) 15-items [49]	The GDS is used to assess an older person's level of depression with simple yes/no response set [59], and the fifteen item screening test has been reported to be satisfactory [49].
	
	Depression Anxiety and Stress Scale 21(DASS) [60]	21-item self-report measure of severity of depression, anxiety, and stress psychological symptoms. Overall Distress can be calculated by summing each of the sub-scale scores with possible scores ranging from 0-63, with higher scores indicating higher distress [48].
	
	
	Scale of Psychological Wellbeing (SPWB) [47]	The SPWB measures well-being and psychological functioning includes six subscales: autonomy; environmental mastery; personal growth; positive relations with others; purpose in life; and self-acceptance [47]. Participants are required to rate agreement on a six point agreement scale, with higher scores indicating greater wellbeing.
	
	Duke Social Support (DSS)[50]	The DSS is used to assess perceived adequacy and size of social support network on a 3 point scale with higher total scores reflecting higher levels of social support [50].

Quality of life	Life satisfaction Scale (LSS)[44]	This single item 7 point delighted-terrible rating scale provides a gestalt measure of life satisfaction, and can yield reliable and valid data [61].
	
	Physical and Mental Health Summary Scales (SF36)^®^	The Physical & Mental Health Summary Scales include eight generic health concepts, selected from 40 included in the Medical Outcomes Study (MOS), and MOS researchers selected and adapted questionnaire items and developed new measures for a 149-item Functioning and Well-Being Profile [62] the source for SF-36^® ^items.
	
	Quality of Life Scales (QoLS)[46]	This 16 item 7 Likert type delighted-terrible self report scale measures satisfaction with five conceptual aspects of life notably material and physical wellbeing; relationships with other people, social, community and civic activities; personal development and fulfilment; and recreation [46].

**Table 5:** Secondary outcome measures continued: physical health and functional status

Outcome Measure	Explanation	Description
Body composition	Anthropometrics	Standing height body weight, waist circumference are obtained in triplicate after 12 hour fasting and body mass index (BMI)) is calculated as fasting body weight (weight kg/height m^2^).
	
	Bioelectrical Impedance Analysis (BIA)	Whole-body skeletal muscle mass (kg) (SMM)* and fat free mass (kg) (FFM) ** were calculated using the average resistance and reactance values of three sequential BIA measures using the BIA Analyser (RLJ Prizum, S/N: B10875E, Mode; BIA-101S).

Cardio-vascular	Pulse Wave Analysis	Pulse Wave Velocity (PWV) Pulse Wave Analysis (PWA) Heart Rate Variability (HRV) is determined using the SphygmoCor Unit and SphygmoCor software.
	
	Ankle Brachial Index (ABI)	Clinton MI) Ankle-brachial index (mean of dorsalis pedis and posterior tibialis/brachial BP in both arms).
	
	Blood Pressure (BP)	Orthostatic hypotension (OH) Orthostatic Blood Pressure Measurement is taken in a fasted state and after rising from a five minute rest in supine position. Twenty-four hour ambulatory BP monitoring, awake and nocturnal means and circadian rhythm are also obtained.

Exercise Capacity	Muscle strength and endurance	Maximal strength measurement will be obtained using the digital K400 Keiser pneumatic resistance machines (Keiser Sports health Equipments, Inc. Fresno, CA). See **table 7 **for details.
	
	6 minute walk distance (6MWD) [63]	Walking endurance was assessed using the six minute walk test which is a proxy for overall cardiovascular endurance capacity (aerobic capacity) and in the elderly subject it may be determined by muscle strength and endurance, balance, orthopaedic or neurologic abnormalities, and other problems[63].
	
	Aerobic Capacity	Maximal exercise capacity assessed on treadmill walking test (stress test).

Physical performance	Gait speed habitual and fast	Habitual and maximal gait velocities is assessed for 2 m (Ultra-timer: Raymar, Oxfordshire, UK) with the average of two times taken as habitual (CV = 8.7%) and maximal (CV = 7.6%) gait velocity respectively.
	
	Gait analysis (Gait Logger)	Participants walk two walks of 2 minute duration and data is recorded using the gait logger Minion EGaitLogger and downloaded
	
	Isometric handgrip strength	Isometric handgrip strength of the non dominant hand is assessed using a JAMAR handgrip dynamometer (Sammons Preston, Bolingbrook, IL)
	
	Chair stand [64]	This test is used as a proxy for lower extremity power, or the ability to generate high forces rapidly, with participants primarily utilising the hip extensor and knee extensor muscle groups [64].
	
	Static Balance	Static balance is assessed up to 15 seconds in five different positions (feet apart in parallel stance, feet together in parallel stance, half tandem stance, tandem stance, and one legged stance), without the use of assistive device with eyes open. Total static balance is calculated by summing the time recorded for each of the six stances[65].
	
	Tandem walk	Subjects complete a 3 meter forward tandem walk along a marked course with and without a cognitive distracter task (verbal fluency)
	
	Stair climb	The purpose of this test is to climb stairs as rapidly as possible to enable the calculation of Power (Watts). Power is calculated from the formula: P (watts) = (M × D) × 9.8/t Where: M = Body mass (kg), D = Vertical distance (m), t = Time (s) and, D = vertical height of the staircase = height of 1 step in meters × number of steps (if they are all the same height).

*Skeletal muscle mass (SMM) = 0.401(height in cm^2^/resistance in ohms)+3.825 (sex: male = 1; female = 0)+age in years(-0.071) + 5.102 [66]

**Fat-free mass (FFM) = -4.03 + 0.734 (height in cm^2^/resistance in ohms) +0.116(body weight in kg) + 0.096 (reactance in ohms) +0.984 (sex: male = 1; female = 0)[67]

**Table 6:** Secondary outcome measures continued: Health status

Outcome measure	Explanation	Description
Health Status	Habitual Physical Activity level	Daily physical activity, sedentary behaviour and sleep quality and quantity are measured with two Actigraph monitors worn for seven days. The actigraph on the waist measures physical activity and the wrist sleep quality, with data analysed using the ActilifeGTIM (version 2.2.3) and ActiWebClient (version 4.2.2) programs.
	
	Cortisol	Five saliva cortisol samples will be collected using the Salivette (Sarstedt Aktiengessllschaft and Company; Kirschbaum & Hellhammer, 1994) and according to manufacturer instructions. A control 'resting' "Pre" and "Post" samples will be taken before exposure to each 'stressor' condition fasting and upon awakening and 30 minute rest, and a prior to stressor and immediately after the termination of the stressor condition. The cognitive 'stressor' condition is Alzheimer's Disease Assessment Scale (ADAS-Cog) assessment during screening and at 6 months assessment (approximately 45 min), the physical stressor is the second exposure to a set of maximal strength testing (1RM testing) at baseline and 6 mo.
	
	Medical History	Physician complete medical history and physical and neurological examination.

Inflammatory/Anabolic/Deoxyribo-Nucleic Acid (DNA) profile	Serum samples for nutritional, biochemical and hormonal factors, pro- and anti-inflammatory cytokines	A venous blood draw is taken after a 12 hour fast for B12, folate, Thyroid Stimulating Hormone (TSH), insulin, glucose (with calculation of insulin sensitivity and beta−cell function using the HOMA2 Computer Model), Liver Function Tests (LFT), cholesterol (Total, High Density Lipoprotein, Low Density Lipoprotein, Triglycerides), Full blood count, creatinine, albumin, homocysteine, and 25−OH vitamin D level, and a second sample taken to measure specialist markers of inflammation: high sensitivity C−reactive protein (hs−CRP), cytokines InterLeukin (IL)−1b, IL−6, IL−8, IL−10, IL−12p70, IL−18 and Tumor Necrosis Factor− proteins (TNF-proteins), as well as Brain Derived Neurotrophic Factor (BDNF), Insulin Growth Factor-1 (IGF-1). At Baseline only a blood draw is taken for genetic Testing for Apolipoprotein allele 4 (APOE 4).

**Table 7:** Secondary outcomes continued: neuroimaging

Outcome	Measure	Description
Structural MRI: T1-weighted Whole Brain Measures	1. Voxel Based Morphometry	A combination of different software packages will be used for automated and semi-automated computational neuroanatomical analyses, in addition to expert manual tracing of hippocampus and entorhinal cortex.
	2. Cortical thickness	
	3. Whole brain volume	
	4. GM volume	
	5. WM volume	
	6. CSF volume	
Regional Measures	1. Automated regional cortical volume measures	
	2. Manually traced subcortical volumes	

FLAIR-weighted MRI	White matter hyperintensity volume	Automated measure of white matter disease load

Magnetic Resonance Spectroscopy (MRS) Relative measures of :	1. N-acetylaspartate (NAA)	Measures of different brain metabolites using MR spectroscopy in the hippocampus and posterior cingulate grey matter.
	2. Cholines	
	3. Myo-inositol	
	4. Creatine+Phosphocreatine	
Resting State functional MRI	1. Bilateral hippocampal connectivity	Seed-based correlational analysis and Independent Component Analysis will be used to characterise individuals' resting state BOLD time series.
	2. Hippocampus functional connectivity map	
	3. Posterior cingulate functional connectivity map	
	4. Default Mode Network (DMN)	
	5. DMN-anticorrelations	

#### Primary outcomes

Cognitive function is measured via the Alzheimer's Disease Assessment Scale-Cognitive (ADAS-Cog) [25], and capacity to perform daily tasks by the Bayer-Activities of Daily Living (B-IADL) [26] which has been found to differentiate between normal ageing and mild to moderate dementia [39].

#### Secondary Outcomes

##### Cognitive function

Global cognitive function is assessed via the Clinical Dementia Rating scale (CDR) [28], and Mini-Mental Status Examination (MMSE) [29]. Specific cognitive functions are assessed by Trail Making Test A and B [40], Matrices and Similarities subtests of the Wechsler Adult Intelligence Scale 3^rd ^Edition (WAIS-III), Symbol Digit Modalities Test (SDMT) [41], Logical Memory I and II subtests of the Wechsler Memory Scale 3^rd ^Edition (WMS-III), Benton Visual Retention Test-Revised 5^th ^Edition (BVRT-R) [42] and verbal fluency (Controlled oral words association test, and animal names). Subjective perception of memory capacity is assessed by the Memory Functioning Scale of the Memory Awareness Rating Scale (MARS-MFS) [43]. Cognitive domain scores will be calculated on the basis of sum of z-scores of index tests, referenced to whole-group baseline results.

##### Psychosocial and quality of life

Psycho-social wellbeing and quality of life are assessed via the Life Satisfaction Scale (LSS) [44], Physical and Mental Health Short-36 (SF-36>)[45], Quality of Life Scales (QOLS) [46], Scale of Psychological Well Being (SPWB) [47], Depression, Anxiety, Stress Scales (DASS) [48], the Geriatric Depression Scale (GDS) [49], and Duke Social Support (DSS) [50].

##### Physical status and level of functional capacity

Physical status and exercise capacity are assessed across seven domains: body composition; cardio vascular profile; exercise capacity; functional performance; nutritional status; health status; and inflammatory and anabolic profile, with measures described in Table 4.

##### Neuroimaging

MRI data are acquired at baseline, 6 months follow up and 18 months follow up, using a 3.0-Tesla Philips Achieva System (see Table 7). For each time point, brain structure is assessed using a T1-weighted whole brain scan (sequence: T1TFE; TR/TE: 6.39/2.9 ms; slice thickness 1.0 mm without gap; field of view: 256 × 256; resolution 1 × 1 mm) and a T2 FLAIR scan (sequence: TIR; resolution: 0.488 × 0.488 × 3.5 mm; TR/TE = 10,000/110 ms). ^1^H-MRS follows in two volumes of interest: left hippocampus (20 mm M/L, 15 mm D/V, 30 mm A/P, oriented along the hippocampus) and posterior cingulate grey matter (20 mm M/L, 20 mm D/V, 20 mm A/P) using the PRESS sequence (TE/TR = 30/2000 ms, 1024 points, 256 averages). Finally, a resting state functional MRI is conducted using T2* echo-planar BOLD sequence (TR/TE = 2000/30 ms, 200 volumes) with the subject's eyes closed.

### Covariates

Covariates specified *a priori *are age, gender, educational history, occupational history, burden of chronic disease (medications and diagnoses), nutritional supplements, history of weight loss in past year and habitual physical activity level.

### Statistical Analysis

We will use an intention to treat (ITT) analytic strategy as our primary analytic treatment of the data. However, we acknowledge the potential bias of any method of imputation [last observation carried forward (LOCF), mean of group, expectation maximization algorithm (EM)] or restriction to observed cases (complete case analysis). Therefore, we will make all attempts to retrieve data from dropouts by obtaining final measures regardless of intervention participation or compliance, and will use the EM method for data missing at random. In addition, we will compare characteristics of dropouts to completers and perform secondary sensitivity analyses (completers, and per-protocol analyses) to examine potential for dropouts and imputation to bias the results.

Mixed modelling of 6- and 18-month outcomes, adjusted for baseline values and any potential confounders identified will be constructed to test our primary and secondary hypotheses. We will test for main effects of CT and PRT, as well as for the interaction term (CT × PRT) to identify isolated and combined training arm significance and effects sizes. Relationships of interest and risk factors for changes in cognitive function and other secondary outcomes will be analysed with simple and multivariate linear and logistic regression models as appropriate. Weighted mean differences, 95% Confidence Intervals and Effect Sizes will be calculated for all outcomes, and clinical meaningfulness will be assessed in light of available data on the expected annual rates of change in this cohort for all known primary and secondary outcome variables.

## Results

We originally estimated the need for a sample size 10% larger than our expected effect size required (n = 132). With our retention rate of >90% to date our recruitment target remains appropriate 120/.90 = 133. Thus, we have recruited 80/133, 60% of our planned cohort to date. Compliance with training sessions to date has been high for all groups with median compliance ranging from 78.44% for sham physical/CT to 100% for PRT/CT. Furthermore there has been one adverse event reported thus far (one rotator cuff injury managed conservatively) in PRT group, and no adverse events during assessments, CT, or sham interventions.

## Discussion and conclusions

Recruitment of subjects has been challenging, with less than 4% of contacts recruited, and 12 telephone screening interviews required for each subject enrolled. However, the most common reasons for ineligibility have been lack of cognitive impairment, or being too physically active, rather than medical exclusions, attesting to the potential generalizability of this volunteer sample to typical older adults with multiple stable chronic illnesses and mild cognitive impairment.

Our primary outcomes of global cognitive status and functional independence are anticipated in 2012. This information will provide novel and robust evidence for the efficacy of cognitive and strength training on cognition and functional status in at risk older adults. This study conforms to all CONSORT criteria for the reporting of RCTs, making it relatively unique in the field to date. Furthermore, the SMART trial will provide valuable information on the persistence of training benefits after cessation of training at 18 months. Recording participant social and recreational activities will also enable SMART to examine the impact of training post intervention on leisure activity, and control against potential confounding effects of participants independently pursuing physical exercise and mental activities.

Our secondary outcomes will enable the first comprehensive investigation of the relative and combined benefits of physical and cognitive training on brain morphology and function, anxiety and depressive symptoms, self-efficacy, quality of life, body composition, cardiovascular risk profile, aerobic and musculoskeletal fitness, and metabolic health. These outcomes will not only provide evidence of the potentially broad benefits of the SMART interventions in this cohort, but also clarify the hypothesized mechanisms contributing to any observed cognitive outcomes.

In summary, SMART will test a non-pharmacological preventative intervention that targets older adults at high risk of cognitive decline. By implementing a regime of physical and mental exercise, we aim to empower the individual, contribute to their physical, cognitive and psychological health, and ultimately improve quality of life.

## Competing interests

The authors have no conflicts of interest. MV has previously received honoraria for speaking at Pfizer sponsored events. HB is an investigator for Pfizer, Novartis, Janssen, Lundbeck, Lilly, a sponsored speaker for Pfizer, Novartis, Janssen, and on advisory Boards for Pfizer, Novartis, Janssen, and Lundbeck. NG holds shares in HeadStrong Brain LLC New York although no dividends, gifts or royalties have ever been received and no work has been conducted for the company since 2007.

## Authors' contributions

All authors critically reviewed the manuscript. NG manuscript draft; HB, PS, MV, MFS, BTB, NG, MB design of study; MFS, HB, PS, BTB, MV, study conception; NJ subject recruitment, telephone screening and scheduling; NG, MFS eligibility screening; NG cognitive assessment; MB, GW, DW, NJ physical assessment; MFS, YW, NJ database and protocol management; MB, NF, NJ, GW training; MV, CS fMRI; NJ, MB, DW, GW, NG data management and analysis.

## Pre-publication history

The pre-publication history for this paper can be accessed here:

http://www.biomedcentral.com/1471-2318/11/19/prepub
